# Multi-level model for the investigation of oncoantigen-driven vaccination effect

**DOI:** 10.1186/1471-2105-14-S6-S11

**Published:** 2013-04-17

**Authors:** Francesca Cordero, Marco Beccuti, Chiara Fornari, Stefania Lanzardo, Laura Conti, Federica Cavallo, Gianfranco Balbo, Raffaele Calogero

**Affiliations:** 1 Computer Science Department, University of Turin, Corso Svizzera 185, Torino 10149 Italy; 2 Molecular Biotechnology Center, University of Turin, Via Nizza 52, Torino 10126 Italy; 3 Faculty of Information and Communication Technology at Rabigh, King Abdulaziz University, Jeddah, Saudi Arabia

## Abstract

**Background:**

Cancer stem cell theory suggests that cancers are derived by a population of cells named Cancer Stem Cells (CSCs) that are involved in the growth and in the progression of tumors, and lead to a hierarchical structure characterized by differentiated cell population. This cell heterogeneity affects the choice of cancer therapies, since many current cancer treatments have limited or no impact at all on CSC population, while they reveal a positive effect on the differentiated cell populations.

**Results:**

In this paper we investigated the effect of vaccination on a cancer hierarchical structure through a multi-level model representing both population and molecular aspects. The population level is modeled by a system of Ordinary Differential Equations (ODEs) describing the cancer population's dynamics. The molecular level is modeled using the Petri Net (PN) formalism to detail part of the proliferation pathway. Moreover, we propose a new methodology which exploits the temporal behavior derived from the molecular level to parameterize the ODE system modeling populations. Using this multi-level model we studied the ErbB2-driven vaccination effect in breast cancer.

**Conclusions:**

We propose a multi-level model that describes the inter-dependencies between population and genetic levels, and that can be efficiently used to estimate the efficacy of drug and vaccine therapies in cancer models, given the availability of molecular data on the cancer driving force.

## Background

Systems biology is increasingly used to get insights into the functioning of complex biological networks. Specifically, the use of mathematical formalisms to investigate the mechanisms affecting tumor growth and maintenance upon vaccination or drug treatment might represent a powerful instrument to efficiently guide the design of long and expensive in vivo experiments [1].

Building network models that accurately represent either biochemical pathways, cell-to-cell interactions, or regulation networks is necessary for different purposes. Indeed, a model provides the basis for a clear description of the interactions involved in a biological system. However, to be useful, a model must be precise and suitable for an analysis that helps in getting a better understanding of the phenomenon under investigation and appropriate formalisms must be used to achieve this goal. Furthermore, when the objective of the study is the behavior of a biological system described at the level of a biochemical reaction scheme, the completion of the modelling process sets the ground for a sensitivity analysis of the model where, at the level of molecule concentrations, it is possible to perturb the net representation or the reaction rates to study the influence of specific elements of the network on the overall functionality of the system. From a structural point of view, a qualitative analysis of the model can be used to select key elements that may suggest interesting features of the experimental system which are worth of detailed investigations (e.g. therapeutic targets). All these points highlight the need of a strong integration between computational modeling and quantitative experimental data. The paper by Kreeger and Lauffenburger [2] reports examples of recently proposed integrations of this type in the field of cancer systems biology. However, even thought a pathway-centric approach is widely and successfully used to investigate cancer in terms of molecular effects, when a specific gene or protein is identified to make a contribution to pathology, it is not easy to determine how its influence is propagated at population level, unless the interaction between molecular effects and population dynamics is specifically addressed by the model.

In this paper we propose a new approach, which allows to describe in a single multi-level model different dynamics levels (e.g. molecular regulation network and cell population) of a complex biological system providing a way of highlighting the interactions among different levels and making easier the model parameter definition. For instance the difficulty of defining the parameters of a population model, in the presence of few measured results, can be overcome deriving such parameters directly by a molecular network that mimics the most relevant biochemical reactions occurring into a cell population, thus accounting for the presence of environmental changes, mutation, and noise in intracellular biochemical reactions.

Even if it is true that with this methodology, dealing with a lack of information is only moved one level down in the modeling process, (functionally) simpler parameters are now used in the basic model of our case study, leaving to the solution of the molecular model the burden of deriving the complex representations of the proliferation parameters that are now allowed to be expressed with intricate functions of time.

As we just said, our case study has been modeled with a 2-level representation. The first level describes regulation aspects of proliferation considering gene interactions; specifically, our experimental model of carcinogenesis is driven by ErbB 2 [3]. This molecular network is designed using the Petri Net [4] formalism which is quite suitable to build models of this type and which allows to compute qualitative and quantitative properties of the experimental system with numerical and analytical methods. Moreover, PNs offer the possibility of representing a reaction scheme as a graphical diagram that supports the comprehension of the behavior of the real system with simple to understand, yet precise descriptions.

The second level describes the population interactions in the ErbB 2-driven carcinogenesis, and is based on the model presented in Fornari's paper [5], where a system of ODEs was used to describe the progression of malignant tumors, assuming the validity the CSC theory [6]. Our ODE model takes into account the main properties of CSCs: tumorigenic capacity, self-renewal, and differentiation into non-stem cells. The hierarchical organization of the tumor is guaranteed both from the growth and progression as well as from the differentiation capacity characterizing CSC subpopulations. CSCs give rise to committed Progenitor Cells (PCs) characterized by a rapid proliferation rate. PCs are able to completely differentiate into Terminally differentiated Cells (TCs). In the paper by Fornari et al., parameters characterizing the behavior of proliferation, death, and differentiation of tumor cell populations are assumed to be affected by external events such as vaccination or pharmacological treatments and are tuned using experimental data based on the tumor mass growth trend observed in mice after a subcutaneous injection of cancer cells. However, to make such cellular model interesting for the biological community, it is necessary to link proliferation and differentiation parameters to the molecular events which control them, thus allowing the visualization at cellular level of perturbations made on the underlying molecular network.

To investigate how the perturbation at molecular level impacts on population models, we used a simulative approach to show the effects of well known inhibition of progression of multifocal preneoplastic lesions [7] in ErbB2-driven carcinogenesis, by means of chronic vaccination and we provide an example of new hypothesis that can be generated using such models and subsequently validated with biological experiments.

## Results and discussion

In this section, we first discuss the new proposed approach in details, and then we show how it can be used to study and analyze the effects of vaccination on a carcinogenesis driven by ErbB 2 [3] receptor family considering both population and molecular aspects.

## Workflow

**Figure 1:**
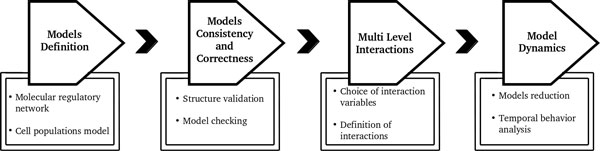
**Workflow**. Organization of the method partitioned in four main steps.

In this paper we propose a new **multi-level approach **to model and analyze complex biological systems, which exploits specific interdependencies among different levels. The overall organization of the method is summarized in Figure 1, and consists of the following four main steps: (1) model definition, (2) model consistency and correctness validation, (3) multi-level model interactions, (4) model dynamics.

### Model definition

In this first step the biological system is represented by a multi-level model, where the number of levels is chosen according to the phenomenon under study. As already highlighted in the background Section, focussing at each level on different aspects of the problem under study, model creation and parameterization are made easier. For instance, our case study which is concerned with the carcinogenesis driven by ErbB 2, was modeled by 2-level model, where the former level describes the molecular regulatory network and the latter one the cell populations. In details, the dynamics of the low level, namely at the gene and molecular scale, is modeled using the PN formalism. It provides an explicit and intuitive representation of the signaling cascade controlled by the ErbB receptors family, capturing the relevant biochemical reactions involved in the regulation aspects of proliferation. Instead, the dynamic of the high level system, namely at the cell population scale, which describes the interactions between different sub-populations of cells is represented by a system of ODEs, following a trend that has already been established in the literature [8].

### Model consistency and correctness

This second step that naturally follows the model definition phase is focused on the validation and verification of the accuracy and correctness of the representation. Among several methodologies, used to perform these analysis, we exploit structural validation and model checking. The model structure is validated using the P-semiflow analysis, and thus identifying the set of places where a given kind of correlated matter is preserved during the evolution of the model in order to make sure that mass conservation laws are respected. On the other hand, model checking is used to verify the consistency and correctness of the model with respect to well-known properties found in the literature and expressed through Computational Tree Logic (CTL), a type of temporal logic. In our case study, model checking is used to verify if the growth factors stimulation always leads to a production of specific protein complexes. Details on how model checking is used in our case study also to analyze other properties of the model will be provided at some length in the Methods Section of this paper.

### Multi level model interactions

After the creation and the validation of models, it is necessary to define how models interact. In our case study, to link populations proliferation parameters with regulation events we identify for each level a set of *interaction points*. Specifically, for the molecular level we select a set of (PN) places playing a pivotal role in cell proliferation corresponding to Bad, cyclinD, and NF-kB proteins. On the other hand, at population level, we select proliferation rates of CSC and PC as the parameters that mostly depend on biochemical reaction dynamics. Hence, we specify interactions defining each proliferation rate as the product of three functions representing the temporal behaviors of protein targets.

### Model dynamics

The last step is related to the analysis of the global model dynamics. First, the corresponding ODE system is automatically derived from the PN model.

In general, this system of ODEs can be very large and complex, thus a preliminary reduction phase is performed to obtain a suitable system of ODEs. Specifically this phase consists in a downsizing of the ODE number by identifying those equations which are redundant using PN structural properties such as P-semiflows. Indeed, we derive a set of ODEs from the PN model and, then, for each minimal P-semiflow of the system one equation from the ODE system is removed. After having reduced the complexity of the model, the temporal dynamics of the quantities contained in the places which play a pivotal role in cell proliferation are studied through numerical integration of the derived ODE system. Hence, the obtained quantities are used as parameters in the ODE system modeling the cell proliferation, and it is solved by numerical integration.

Referring to our case study, in the following paragraphs we show how our methodology can be be put in practice discussing first the individual components of our two-level model.

### First level: molecular regulation network

**Figure 2:**
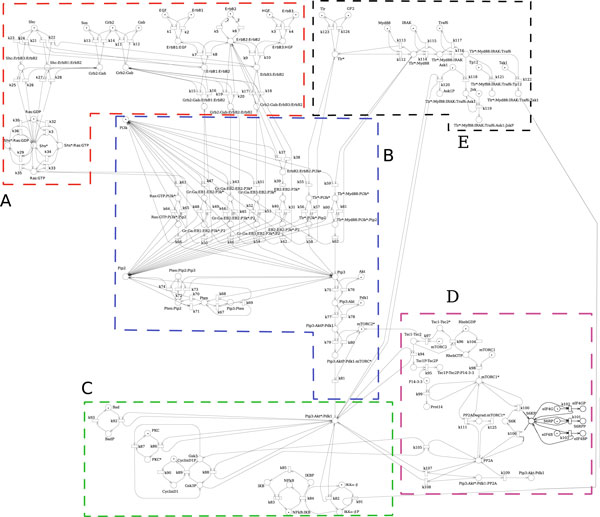
**Petri Net of molecular model**. PN representing the 111 biochemical reactions describing regulation aspects of proliferation model.

**Figure 3:**
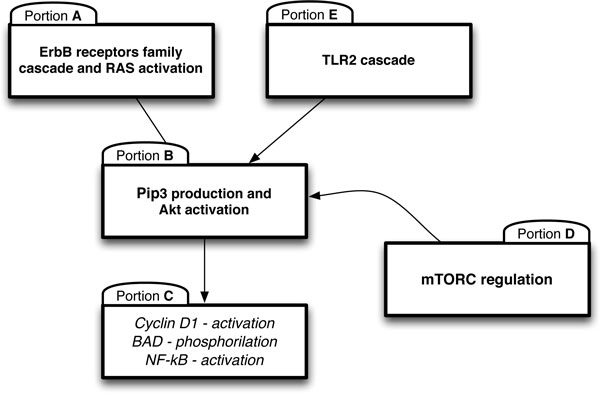
**Simplified schematic representation of molecular network**.

The network provides a fairly complete representation of the signaling cascade controlled by the ErbB receptor family where the targets are represented by three proteins (cyclinD, NF-kB and BAD) playing a pivotal role in cell proliferation. The temporal behaviors of these targets are used to control the proliferation rates of CSC and PC populations. The main effect of ligand binding and dimerization of ErbB family receptors regards the activation of the phosphoinositol-3-kinase(PI3K)/Akt pathway. Akt is a serine/threonine kinase that functionally modulates numerous substrates involved in the regulation of cell proliferation, survival, angiogenesis and tissue invasion [9]. This signaling cascade is modeled using a Petri Net formalism (see Methods for more details). Figure 2 shows the complete PN formed by 111 proteins (places) and 124 reactions (transitions) which require the definition of 235 parameters (reactions and parameter values are available in the Additional file 1 and in Additional file 2). The model allows to specify the temporal dynamics of protein targets and to investigate how therapeutic approaches, such as vaccination or drugs treatments, impact and spread on molecular network. A high level description of our molecular network is reported in Figure 3, where it is highlighted its organization in 5 portions: (A) ErbB activation cascade; (B) Phosphatidylinositol 3,4,5-triphosphate, Pip3 production and Akt activation; (C) downstream effects of Akt activation; (D) mammalian target of rapamycin, mTOR, regulation, and (E) Toll-like receptor 2, TLR2, cascade. Portion A describes the ligand binding and dimerization for three ErbB receptors: ErbB 1, ErbB 2 and ErbB 3. These reactions are based on the results contained in the paper by Birtwistle [8] which describes a quantitative kinetic model of the ErbB family cascade to the Akt activation. ErbB receptor ligands, EGF and HGR, activate ErbB 1 and ErbB 3, respectively. Both the ligand-bound ErbB 1 and ErbB 3 dimerize with receptor ErbB 2 and then lead to the recruitment of different adapter proteins, namely Grb2, SOS and Gab1. On the other hand, no known ligand binds ErbB 2: it is a distinguished member of the ERBB family since it does not bind any of the known ligands with high affinity, but it is the preferred heterodimeric partner for other receptor of ErbB family. The downstream signaling of ErbB receptors, conditional on the recruitment of adapter proteins, involves the activation of the enzyme PI3K. Ligand-bound receptors after dimerization with ErbB 2, can recruit Shc, via Grb2/Sos complexes. This event is mutually exclusive with respect to the activation of PI3K. The Shc adapter is involved in the Ras/MAP kinase pathway, resulting, specifically, in GDP/GTP exchange and Ras activation. Also Ras is subsequently involved in the PI3K/Akt pathway.

The main effects of the downstream signaling is the production of Pip_3 _that leads to the activation of Akt, as reported in portion B of the network. The production of Pip_3_, which is a second messenger involved in the regulation of different processes, is catalyzed by PI-3K starting from Phosphatidylinositol 4,5-triphosphate, Pip_2_. In portion B a set of reactions involved in the regeneration of Pip_2 _is also reported. Its recovery results from the contribution of the Pten-dependent dephosphorylation of Pip_3_.

With respect to Birtwistle's work, we extend the network with three additional blocks (Portions C, D, and E). Portion C describes the downstream effects of Akt activation. Akt has a critical regulatory role in many cellular processes, and in particular in cancer progression. As described before, we decided to focus the effects of Akt on three targets:

• *the transcription factor Bad *the proliferation action of Akt is mediated through the direct inhibition of this pro-apoptotic signal,

• *the activation of cyclinD *Akt occurs at the G1-S transition of the cell cycle via phosphorylation and inhibition of glycogen synthase kinase 3-beta (GSK-3*β*) that stabilizes cyclin D1,

• *the transcription factor nuclear factor-kappa B, NF-kB *Akt promotes NF-kB activity since it directly phosphorylates I-kappa-B kinase *α*, IKK*α*, to activate NF-kB whose broad oncogenesis activity - through its ability to control cell proliferation and to suppress apoptosis - is well known.

Another important regulation of cell growth by Akt regards its primary effect on mTOR whose action is depicted in portion D of the network. mTOR is associated with two complexes: the rapamycin-sensitive TORC1 complex which controls S6K phosphorylation and 4E-BP1 to regulate translation, and TORC2 that controls the phosphorylation of Akt. The activation of TORC1 by Akt involves the phosphorylation of TSC2, which reveals a negative regulatory effect on mTOR controlled by the GTPase Rheb.

Finally, portion E specifies the cascade of TLR2. Functional analysis of mammalian TLRs has revealed that they recognize specific patterns involved in the cell proliferation. The signaling pathway via TLR2 recruits the adapter protein MyD88. Upon stimulation, MyD88 recruits IL-1 receptor-associated kinase (IRAK) to TLR2. IRAK is activated by phosphorylation and then associated with TRAF6, leading to the activation of two distinct signaling pathways, and finally to the activation of JNK and NF-kB.

Overall, this network is a modification of that proposed by Birtwistle in order to account the characteristics of preclinical breast cancer model based on BALB/c mice transgenic for the transforming rat ErbB2 oncogene, BALB-neuT mice. BALB-neuT mice develop breast cancer with 100% penetrance [10]. These animals are transgenic for a mutated ErbB2 rat gene, encompassing a single point mutation that replaces the valine residue at position 664 in the transmembrane domain with glutamic acid favoring ErbB2 homo-dimerization thus transforming the ErbB2 proto-oncogene into a dominant transforming oncogene [11]. In vivo experiments have shown that PI3K represents an important element in the ErbB2 signal transduction since anti-ErbB2 antibodies impair PI3K/Akt-mediated tumorigenic effects [12], these experiments also demonstrate the ability of ErbB 2 to activate directly Akt without the involvement of growth factors. Moreover, the choice of adding the TLR2 contribution to the proliferation pathway derives from the observation that the TLR2 receptor shares the PI3K activation network [13] with ErbB2 [14], and accounts for recent results that show TLR2 to be expressed by breast cancer cell lines [15] and to be involved in cancer invasiveness.

### Second level: cell population model

We have investigated the proliferation of the three tumor cell populations CSC, PC and TC using an ODE based model. The system of ODEs presented in Fornari's paper has been modified in this work neglecting (at this level) the therapy effects. The resulting model of the dynamics of these cancer cell populations is constructed by specifying the following system of linear and homogeneous ODEs:

(1) $$\begin{array}{llll} & \frac{dNcsc}{dt}\;=\;P_{sy}\omega_{CSC}N_{CSC}+\gamma_{PC}\overset{3}{\underset{j=1}{\sum }}N_{PC_{j}}-\eta_{1}N_{CSC}-d_{1}N_{CSC}\; & & \;\\ & \frac{dN_{PC_{1}}}{dt}\;=\;P_{asy}\omega_{CSC}N_{CSC}-\omega_{PC}N_{PC_{1}}-\gamma_{PC}N_{PC_{1}}-\eta_{1}N_{CSC}-\eta_{2}N_{PC_{1}}-d_{2}N_{PC_{1}}\; & & \;\\ & \frac{dN_{PC_{j}}}{dt}\;=\;2\omega_{PC}N_{PC_{j-1}}-\omega_{PC}N_{PC_{j}}-\gamma_{PC}N_{PC_{j}}+\eta_{2}N_{PC_{j-1}}-\eta_{2}N_{PC_{j}}-d_{2}N_{PC_{j}}\;j=\;2\ldots 3\; & & \;\\ & \frac{dN_{PC_{i}}}{dt}\;=\;2\omega_{PC}N_{PC_{i-1}}-\omega_{PC}N_{PC_{i}}+\;\eta_{2}N_{PC_{i-1}}-\eta_{2}N_{PC_{i}}d_{2}N_{PC_{i}}\;\;i=\;4\ldots 6\; & & \;\\ & \frac{dN_{PC_{7}}}{dt}\;=\;2\omega_{PC}N_{PC_{6}}+\eta_{2}N_{PC_{6}}-\;\eta_{3}N_{PC_{7}}-d_{2}N_{PC_{7}}\; & & \;\\ & \frac{dN_{TC}}{dt}\;=\;\eta_{3}N_{PC_{7}}-d_{3}N_{TC}\; & & \;\\ & \; & \end{array}$$

where N*_CSC_*, $N_{PC_{i}}$ and N*_TC _*are the numbers of cancer stem cells, progenitor cells, and terminal cells, respectively. Notice that the terms characterizing these equations depend on 4 parameters: *ω_CSC _*and *ω_PC _*describe the proliferation rates; *γ_PC _*represents the bidirectional inter-convertibility parameter that involves CSC, *PC*_1_, *PC*_2 _and *PC*_3 _subpopulations; *d_i _*indicates the death rate; and *η_i _*describes the differentiation rates.

### Parameter definition

Before discussing how model consistency and correctness are validated in our case study we explain how the input parameters for the two models are chosen. All the reaction rates of the molecular network as well as the initial protein concentrations, reported in the Additional file 2, are tuned starting from the values reported in the Birtwistle's paper. For what concerns TLR2 receptor concentration, we evaluated the presence of surface expression of TLR2 in TUBO cell line, which is a cell line derived from BALB-neuT tumors [16]. TLR2 positive cells represent a significant subpopulation of the ErbB2 positive cells, which constitute the cell majority both in the TUBO cell line (E) and in the serial passages of spheroids formation (P1, ..., P3). Interestingly, TLR2 positive cells increase over serial passages of spheroids formation, which represent a method to enrich in CSC, see Table 1. For this reason, we set the concentration of TLR2 in CSC subpopulation 100 times higher than that of PCs.

**Table 1:** TLR2 and Neu expression on TuBo (E) and serial passages mammospheres (P1, P2, P3).

Cell line	ErbB2 (%)	TLR2 (%)
E	52.7 ± 4.2	8 ± 1
P1	51.9 ± 12.7	21 ± 3
P2	45.5 ± 10.5	24.5 ± 5.3
P3	41.2 ± 10.4	27.1 ± 3.8

Percentage of positive cells evaluated by FACS analysis. Data shows mean +/-SD from 6 different experiments.

The initial marking of three growth factors is defined as a function which models injections happening at regular time intervals. For what concern the parameters at population level, the TCs/CSCs ratio was defined indirectly on the basis of spheroids that can be produced starting from a cell culture of 1000 TUBO cells. Since spheroids are clonal, i.e. each spheroid is derived from a unique CSC, counting the number of spheroids allows to quantify the number of CSCs present in the starting cell culture (15 +/-5 CSCs every 1000 TUBO cells). The differentiation, death, and bidirectional inter-convertibility parameters are set as reported in Fornari's work [5]. Otherwise, proliferation rates of cell population are defined considering several proteins dynamics at regulation level.

#### Model consistency and correctness

The consistency and correctness of the model has been verified applying three preliminary checks. The first check is based on P-semiflows that, as explained in details in the Methods Section, can be used to identify the sets of places where a given kind of correlated matter is preserved. In this way using the biological reactions involved in the model construction it is possible to identify sets of places that must appear in the same P-semiflows.

For instance if we consider the following reactions:

$$\begin{array}{ccc} R\text{:}41 & Pip3+Pten & \underset{k_{68}}{\overset{k_{67}}{↔}}Pip3:Pten\\ R\text{:}42 & Pip3:Pten & \overset{k_{69}}{\underset{}{\to }}Pip2+Pten\\ R\text{:}43 & Pten+Pip2 & \underset{k_{71}}{\overset{k_{70}}{↔}}Pten:Pip2\\ R\text{:}44 & Pten:Pip2+Pip3 & \underset{k_{73}}{\overset{k_{72}}{↔}}Pten:Pip2:Pip3\\ R\text{:}45 & Pten:Pip2:Pip3 & \overset{k_{74}}{\underset{}{\to }}Pten:Pip2+Pip2 \end{array}$$

we observe that the places representing the conservation of Pten are correctly in the same P-semiflow: Pten, Pip_3_:Pten, Pip_2_:Pten, Pten:Pip_2_:Pip_3_.

The second check is based on model checking: a technique that provides a useful quality control for the development of large scale complicated models. As described in the Methods Section, model checking is based on the use of temporal logic to specify system behavioral proprieties which can be processed automatically using computationally efficient procedures that determine whether they are effectively reproduced by a model. In this work we have used model checking to verify the consistency and correctness of our model with respect to a set biological reactions used for its construction, and to other well-known properties found in literature.

For instance, the following reachability query has been tested and shown to be satisfied in our model: the growth factors stimulation always leads to a production of at least one of the following protein complexes: HER2:Pi3k, TLR:Pi3k, TLR:MyD88:Pi3k, H2:Gr:Gab:Pi3k, Ras:GT:Pi3k, ERBB3:ERBB2:Gr:Ga:Pi3k, and ERBB1:ERBB2:Gr:Ga:Pi3k

$$\begin{array}{c} EF(\left(HER2^{*}:Pi3k^{*}>0\right)\vee \left(TLR:Pi3k^{*}>0\right)\vee \left(TLR:MyD88:Pi3k^{*}>0\right)\vee \vee \left(H2:Gr:Gab:Pi3k^{*}>0\right)\\ \vee \left(Ras:GT:Pi3k^{*}>0\right)\vee \left(RERBB3:ERBB2:Gr:Ga:Pi3k^{*}>0\right)\vee \left(ERBBI:ERBB2:Gr:Ga:Pi3k^{*}>0\right) \end{array}$$

Moreover, examples of pathway queries which show that our model exhibits one well-known properties discussed in the literature [17] is: mTOR inhibition abrogates feedback inhibition of the proliferation pathway resulting in Akt activation;

$$AG\left(P3Akt^{*}:Pdk1\neq 0\Rightarrow \left(TO2^{*}\neq 0\vee \left(P3:Akt:Pdk1:TO2^{*}\right)\right)\right)$$

An example of steady-state property that has been proved in our model is: the system exhibits a cyclic behavior with respect to the presence of Pten

EG((Pten⇒EF¬Pten)∧((¬Pten⇒EFPten).

Finally the last check consists in verifying that the quantitative behavior of the model is consistent with the literature, for instance according to results presented in [18] we verified that Pten inhibition leads to an inhibition of the proliferation pathway.

### Multi-level model interaction

To link proliferation (population level) with regulation events (regulatory network) it is necessary to set interaction points from both models. Those points of interactions are selected through the singling out of proteins (at regulatory level) directly involved in the phenomenon under study (at population level). As hinted before, for what concerns the population level, we select proliferation parameters, i.e. *ω_CSC _*and *ω_PC _*. Instead, at the molecular level we select proteins which have a pivotal role in cell proliferation, i.e. cyclin D, NF-kB and BAD. The interaction is then defined assigning at proliferation parameters specific values deduced from those target proteins. In detail, three functions representing the temporal behaviors of cyclinD, NF-kB and BAD are created for both CSC and PC regulatory networks. These functions are obtained from the solution of the ODE systems corresponding to the (first level) molecular network of CSC and PC. Proliferation rates are then evaluated as the product of the three functions, which take different values in CSC and in PC regulatory networks.

The vaccination backlash, applied at molecular level, is directly reflected on protein targets.

## Model dynamics

In this last step we describe the two experimental analyses performed within our case study.

### Effects of ErbB2 vaccination

**Figure 4:**
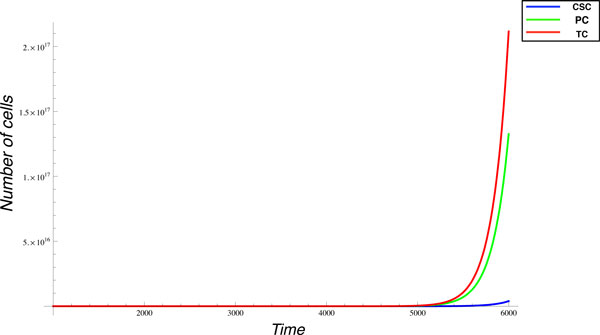
**Breast cancer growth**. Simulation of growth behaviors of CSC, PC and TC subpopulations in the breast cancer growth considering the initial condition of concentration and rate and with three growth factors injections starting from time 1500.

To evaluate if the ErbB2 network controlling cell proliferation exhibits a behavior similar to that observed in BALB-neuT animals (at least from a qualitative point of view), we investigated the effect of ErbB2 repetitive vaccinations on our model. Since ErbB2 is constantly active due to the mutation that favors its homo/hetero-dimerization, without the need of ligand binding, proliferation is always stimulated and it results in a massive production of TCs. On the other hand, the sub-population of CSCs is a small, but very important quantity, that represents the driving force of tumor development as shown in Figure 4. In this experiment, growth factors are injected first at time 1500 and subsequently other two times with intervals of 1000 time units.

**Figure 5:**
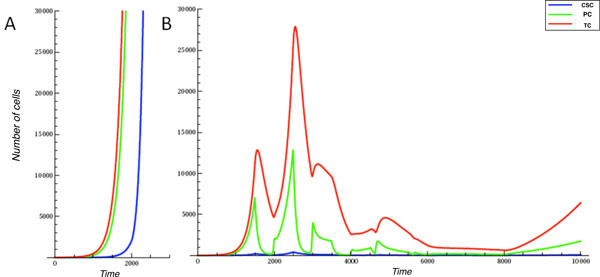
**ErbB2 vaccination on breast cancer**. Panel A reports the exponential increment of TCs and PCs after time 1000. Otherwise in panel B it is applied a vaccination effect on ErbB 2 receptor in five injection spaced every 1000 time units.

**Figure 6:**
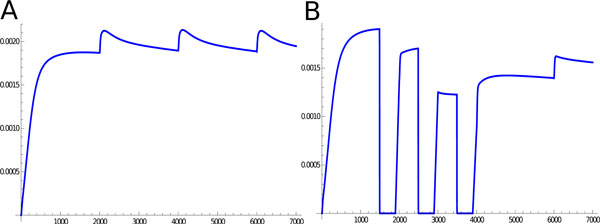
**Cyclin D behavior**. Panel A shows the cyclinD trend in breast cancer growth while panel B reports the cyclinD during three vaccination injections staring form 1500 time units.

We focused on the analysis of this phenomenon to test the validity of our model since chronic vaccination against ErbB 2, in BALB-neuT mice, is well-known and allows progressive clearance of neoplastic lesions and complete protection from the tumor in 1-year old mice [7]. Furthermore, it has been shown that anti-ErbB2 Ab induces a functional block of ErbB2 receptor function [19], down-regulates its expression on the cell membrane [19,20], impedes its ability to form the homo or heterodimers that spontaneously transduce proliferative signals to the cells [20,21], and blocks its ability to bind ligands [22]. Since vaccination directly affects the concentration of ErbB2 on cell surface, it represents a suitable test to establish if our model can simulate a cell growth trend similar to that observed in the BALB-neuT model upon vaccination. Without external interventions, the number of TCs starts to increment exponentially immediately after time 1000, see Figure 5A. In case we apply five vaccination steps, starting just before the exponential growth, i.e. at time 1000, and repeating the vaccination every 1000 time units, the exponential growth is strongly delayed, as shown in Figure 5B. Although our model seems to simulate the effect of vaccination observed in BALB-neuT animals, it must be noticed that BALB-neuT experiments are based on the observation of tumor mass changes, while our model gives a representation at cell population level. Furthermore, our representation is based on an arbitrary time scale, while the BALB-neuT experiments are based on results generated over months. Aligning the time line of the two models corresponds to the difficulty of obtaining reliable experimental estimations of the amount of tumor cell present in tumor mass of a given size (e.g. a tumor of 1 mm of diameter). The growth behavior with and without the vaccination effect is obviously reflected at molecular level. Figure 6A shows the behavior of cyclinD in the cancer growth, it is notable that this dynamics reveals three peaks at times corresponding to the growth factors injections. Figure 6B reflects the cyclinD trend during ErbB 2 vaccination, and shows three definite deeps consistent with the three vaccinations and one bump synchronized at time 6000 with a growth factor injection.

### Involvement of TLR2 in CSC proliferation

**Figure 7:**
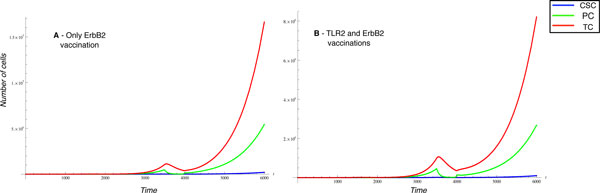
**ErbB2 and TLR2 vaccinations on breast cancer**. Panel A shows the behavior of CSC, PC and TC subpopulation after only a ErbB2 vaccination at 1500 time while panel B reports the combinatorial ErbB2 and TLR2 vaccinations both at 1500 time units.

Although our model is still incomplete and needs further refinements, it successfully provides a proof of concept that the use of molecular networks to estimate specific parameters for the high level ODE systems (performed also using a network perturbation analysis) represents an interesting method for the formulation of new hypothesis to be tested with in vitro/in vivo experiments. As an example of the application of this methodology, consider the case in which we want to evaluate the presence of TLR2 on the surface of breast cancer cell and the effect that it has on the AKT/PI3K network. Preliminary findings suggested to investigate with our model weather the perturbation of TLR2 could functionality affect the CSC driven proliferation in a significative manner. For this reason, we have inserted the TLR2 regulation network as part of the network controlling cell proliferation parameters. Furthermore, on the basis of the above mentioned experimental data, the TLR2 network is only acting on CSC proliferation. We also analyze the effect of a vaccine against ErbB2 and TLR2 at population level. From this experiment we observed a limited reduction of the cell number at the time of vaccination in presence of TLR2 vaccination that is reflected in a reduction of cancer cells in particular TCs. Indeed, if only the ErbB 2 vaccination is performed, it is possible to observe about 10^7 ^TCs at time 6000, (see Figure 7), while if we perform also the TLR2 vaccination at the same time point there are 8*10^6 ^TCs, (see Figure 7B). Such reduction is not sufficient to affect the overall tumor growth, since we did not observed any changes in the time at which the exponential tumor growth starts, with or without TLR2 vaccination.

We are presently evaluating with in vitro experiments, the effect of TLR2 silencing in TUBO cell proliferation to confirm these simulation results. It is notable that literature data [16] indicate that TLR2 mediate innovation by the activation of the NF-kB pathway. This finding together with our observation that TLR2 positive cells are mainly associated with subpopulation of cancer cells enriched for CSC (Table 1), suggest that TLR2 might play some important role in CSC invasiveness. Thus, the TLR2 network might represent an interesting starting point to design a network controlling the parameters linked to CSC from PC differentiation, since invasiveness is associated with undifferentiated cells, i.e. CSC, and is lost in fully differentiated cells, i.e. TC.

## Conclusions and perspectives

In this paper we propose a novel approach in which a multilevel model is constructed and where molecular networks are used to estimate certain parameters of a cell population model based on a system of ODEs. With this method we have been able to reproduce at a qualitative level the effect of anti-ErbB2 chronic vaccination in BALB-neuT model. Although the model needs some refinement to provide a punctual representation of vaccination, i.e. aligning the time line of the computational model with in vivo data, it successfully supports the idea that new in vitro/in vivo experiments can be designed to test hypothesis that are formulated on the basis of the solution of the model. Furthermore, our approach can be extended to consider the immunological tumor micro-environment by adding new equations in the ODE system of the population representation and by defining their parameters on the basis of a cell-to-cell network, instead of a genetic network. This might be particularly interesting in the area of combined treatment development. Tumor vaccination alone is not sufficient to eradicate the disease, but combined with other immuno-pharmacological treatments, affecting the CSC differentiation rate might represent an interesting approach in the area of tertiary cancer prevention, i.e. reducing the negative impact of disease by restoring functions and reducing disease-related complications.

## Methods

The following section reports the details of the biological techniques used for the experiments as well as the notation and the basic definitions of formalism and algorithms used for the analysis discussed in this paper. Part of this section is similar to an analogous description reported in our previous work [23] and is included here only to help the reader in understanding the subsequent discussion.

### Flow cytometry analysis

E, P1, P2, and P3 cells were collected after 7 d of culture and disaggregated through enzymatic and mechanical dissociation. They were then washed in PBS (Sigma-Aldrich) supplemented with 0.2% BSA and 0.01% sodium azide (Sigma-Aldrich) and stained for the membrane antigen Toll Like Receptor (TLR) 2 using an Alexa Fluor647-conjugated anti-TLR2 and for ErbB2 antigen using Ab4 moAb (Calbiochem) followed by FITC anti-mouse IgG (Dako Cytomation) as secondary Abs. Samples were collected and analyzed with a CyAn ADP Flow Cytometer and Summit 4.3 software (DakoCytomation).

### Petri Net formalism

A brief introduction of the PN formalism [24] is provided here to help the reader in understanding the model of the proliferation pathway that we have used in this work.

PNs have been first proposed for the representation of biological pathways by Reddy et al. in [4]. Subsequently, many other researchers discussed the advantages of using PNs to model biological systems [25] because of their ability of representing reaction systems in a natural graphical manner and of their capability of allowing the computation of qualitative and quantitative information about the behavior of these systems. Most of the analysis of PN models in Systems Biology is performed using simulative approaches [26,27] where transitions occur after random firing delays. The extension of the basic PN formalism with stochastic firing delays of this type has been proposed in the literature with the definition of the so-called Stochastic Petri Nets (SPNs) [28-30] which allow the automatic construction of an underlying Continuous Time Markov Chain (CTMC) that can be studied using numerical or simulative techniques and that can also be translated into systems of ODEs when average results are sufficient for the analysis [31]. Several papers recently published by Heiner et al. [32,33] show that the available biological data can be analyzed by means of PNs formalism in order to obtain the general behavioral patterns and timing measures of biological entities. In general, biological models are affected by a high level of complexity due to the functional properties of the systems that are considered. The interaction of qualitative and quantitative analysis is necessary to check a model for consistency and correctness as we will show in the rest of this paper using PNs. In details, PNs are bipartite directed graphs with two types of nodes: places and transitions. The places, graphically represented as circles, correspond to the state variables of the system (e.g. enzymes, compounds, etc.), while the transitions, graphically represented as boxes, correspond to the events (e.g. interactions among biochemical entities) that can induce state changes. The arcs connecting places to transitions (and vice versa) express the relations between states and event occurrences. Places can contain tokens (e.g molecules of the corresponding entities) drawn as black dots within places. The state of a PN, called marking, is defined by the number of tokens in each place. The evolution of the system is given by the firing of enabled transitions, where a transition is enabled if only if each input place contains a number of tokens greater or equal than a given threshold defined by the cardinality of the corresponding input arc. A transition occurrence/firing removes a fixed number of tokens from its input places and adds a fixed number of tokens into its output places (according to the cardinality of its input/output arcs).

The set of all the markings that the net can reach, starting from the initial marking through transition firings, is called the Reachability Set (RS). Instead, the dynamic behavior of the net is described by means of the Reachability Graph (RG), an oriented graph whose nodes are the markings in the RS and the arcs represent the transition firings that produce the corresponding marking changes.

Here we recall briefly the notation and the basic definitions that are used in the rest of the paper.

### Definition: Petri Net

*A PN system is a tuple *$N\;=\;\left(P,T,I^{-},I^{+},{\text{m}}_{0}\right)$*where:*

• *P *= {*p_i_*} *is a finite and non empty set of *places.

• *T *= {*t_i_*} *is a finite and non empty set of *transitions *with *P ∩ T = ∅.

• $I^{-},I^{+}:T\times P\to \mathbb{N}$*are the *input, output*, that define the arcs of the net and that specify their multiplicities*.

• ${\text{m}}_{0}:P\to \mathbb{N}$*is a multiset on P representing the *initial marking *of the net*.

A *marking *m (or state) of a PN is a multiset on *P*. A transition *t *is *enabled *in marking *m *iff *I^-^*(*t, p*) ≤ **m**(*p*), ∀*p *∈ *P *, where **m**(*p*) represents the number of tokens in place *p *in marking **m**. Enabled transitions may *fire*, so that the firing of transition *t *in marking **m **yields a marking **m**' = **m **+ *I*^+^(*t*) - *I*^-^(*t*). Marking **m**' is said to be reachable from **m **because of the firing of *t *and is denoted by **m**[t〉**m**'. The firing of a *sequence σ *of transitions enabled at **m **and yielding **m**' is denoted similarly: **m**[*σ*〉**m**'.

### How to check model consistency and correctness

Before describing how it is possible to study the temporal dynamics of a phenomenon described with a PN model, we discuss two preliminary analysis steps useful to verify the model consistency and correctness, based on P-semiflows and CTL properties. The first step can be used to identify the set of places where a given kind of correlated matter is preserved during the evolution of the model, while the latter allows the modeler to verify more complex behavioral properties.

Mathematically, P-semiflow [34] can be defined as follows:

### Definition: P-semiflow

*Given a Petri Net, let C be the Incidence Matrix whose generic element *$c_{t,p}=I^{+}\left(t,p\right)-I^{-}\left(t,p\right)$*describes the effect of the firing of transition t on the number of tokens in the place p; and let *$x\in Z^{\left|P\right|}$*be a place vector; then a P-semiflow is a place vector x such that it represents an integer and non-negative solution of the matrix equation xC *= 0.

All the P-semiflows of a PN can be expressed as linear combinations of a set of minimal P-semiflows, and the support of a P-semiflow F , denoted *supp*(F) can be defined as the set of nodes corresponding to the non-zero entries of F . Using *supp*(F), each P-semiflow F  allows the computation of a corresponding weighted sum of tokens contained in a subset of places of the net that remains constant through the entire evolution of the model; this constant ia called P-invariant.

In a biological context, where tokens represent compounds, enzymes etc., the interpretation of such P-invariant is relatively simple: the places of *supp*(F) represent the portion of the PN where a given kind of correlated matter is preserved. Obviously when all the places of a net belong to at least one P-semiflow, then the markings of the places are bounded and the state space of the net is finite.

Finally it is important to observe that P-semiflow analysis involves only the structural proprieties of the net and is thus independent of the initial marking of the PN.

The second preliminary analysis step allows the user to verify more complex behavioral properties using the model checking technique, a well-established formal method that is widely-used for ascertaining the correctness of real-life systems. It requires a description of the system usually given in some high level modeling formalism such as PN, and the specification of one or more desired properties of the system, normally using temporal logics. Given this input, the model checker can derive the system behavior (e.g. generating the system RG) and automatically verify whether or not each property is satisfied though a systematic and exhaustive exploration of the RG.

Among the many available temporal logic formalisms, we choose the Computational Tree Logic, CTL, a branching-time logic that extends propositional logic used for describing states, with operators for reasoning over time and non-determinism.

In details the following temporal operators are considered in CTL: *Xp *meaning that the proposition *p *is true at the next transition, *Gp *meaning that *p *is always true, *Fp *meaning finally true, *pUq *meaning that *p *is true until *q *becomes true. For reasoning about non-determinism, the two following path quantifiers are used: *Ap *meaning that *p *is true on all paths and *Ep *meaning that *p *is true on some path. All the temporal operators have to be immediately preceded by a path quantifier, hence *AXGp *is not a valid CTL formula, since the temporal operator *G *is not preceded by a path operator. Moreover, atomic propositions consist of statements on the current token situation in a given place, and they can be recursively composed into more complex propositions using the standard logical operators: ¬ (negation), ∧ (conjunction), ∨ (disjunction), and ⇒ (implication). Hence the CTL can be sufficiently expressive to encode a wide range of biological queries:

• **reachability queries: **there is a cascade of reactions that lead to the production of a protein *p *- *EFp*;

• **pathway queries: **an enzyme can reach an activation state *s *through a substrate-bound state *sb *- *EF *(*sb *∧ *EF *(*s*)); a cell can reach a state *s *without violating a certain constrain *c *- *E*(*cUs*); a protein *p *can be synthesized without a set of transcriptional factors *q *- *E*(¬*qUp*);

• **steady state query **a certain state *s *of a network is a steady state - *s *⇒ *EG*(*s*); an enzyme can stay in active or inactive state - *EF *(*AGs*); an enzyme exhibits a cyclic behavior with respect to the presence of an activator or inhibitor *z *- *EG*((*z *⇒ *EF *¬*z*) ∧ ((¬*z *⇒ *EFz*).

Finally, it is worth noting that, while CTL is an extremely powerful and flexible language to describe special properties, it can be used only by skilled users since it requires a certain experience to correctly express the specification of the desired behavior. Furthermore model checking technique, in case of very complex systems, can require substantial computational resources (in terms of memory and time) since it needs the generation of the RG of the system. In literature several approaches based on efficient RG encoding and manipulation [35] or level concept and monotonic liveness [33] have been proposed to cope with such problem.

### How to analyze temporal dynamics of the modeled system

To model and study the temporal dynamics of a PN we have to introduce temporal specification in the formalism. As we already said at the beginning of this Section, the most common timed extension of PN is Stochastic PN, SPN [28] in which exponentially distributed random delays (interpreted as durations of certain activities) are associated with the firings of the transitions. In details a SPN can be defined as a pair (N,w), where N  is a Petri net and $w:{\mathbb{N}}^{P}\times T\to \mathbb{R}$ is a (possibly marking dependent) function that assigns to each transition of the net the rate of a negative exponential distribution of the firing delay.

Hence, for any transition *t *it is necessary to specify a function *w*(*m, t*), so that when *t *is enabled in a marking *m *then *w*(*m, t*) has to be evaluated to provide the rate of *t *in *m*.

Assuming that the firing times are characterized by probability function with infinite support, this way of adding temporal specifications in the model does not modify the qualitative behaviors of SPN underlying un-timed models so that all the available theoretical results for the PNs can be reused. Specifically, where the firing time distributions is negative exponential, its memory less property allows to recognize that the temporal behavior of the model corresponds to a Continuous Time Markov Chain (CTMC) that can be represented as a graph which is isomorphic to the RG of the same model without time. Then, each marking of the SPN corresponds to a state of the CTMC and the stochastic approach based on SPN adopts a discrete view of the quantity of the entities that appear in the mathematical representation as state components. This means that the temporal behavior of a SPN is seen as a random process governed by the so-called Chapman-Kolmogorov differential equations [36], which corresponds to the behavior of the biological system described by the Master Chemical Equations [37]. However, for very complex model, the underlying CTMC cannot be derived or/and solved due to well-known state space explosion problem. To cope with this problem, the simulative approach can be used to estimate the quantities of interest at the cost of extensive computational efforts [38,39]. Another way of studying this type of model is that of using a so-called deterministic approach in which from an SPN model, it is possible to derive a set of ODEs (one for each place) which assumes that the temporal behavior of the quantity of the entities contained in the different places is a completely predictable process [43]. When modeling metabolic pathways, the most common way to translate the reactions into a set of ODEs is provided by the law of Generalized Mass Action (GMA) [40] from which the system of ODEs describing the model is of the form:

$$\frac{dX_{i}\left(t\right)}{dt}=\overset{N_{i}}{\underset{j=1}{\sum }}w_{ij}\overset{E}{\underset{h=1}{\prod }}X_{h}{\left(t\right)}^{g_{ijh}}\;i=1,\ldots ,E$$

where *E *is the number of interacting entities and *X_i_*(*t*) represents the amount of the *i^th ^*entity at time *t, N_i _*the number of reactions in which the *i^th ^*entity is involved, the parameters *w_ij _*the rate describing the speeds of these reactions, and the parameters *g_ijh _*the so-called kinetics orders which depend on the stoichiometry and on the mechanisms of the reactions. The ODEs and the initial amount of the different entities can be automatically obtained from the SPN representation, and numerical integration of the ODEs is performed to calculate the quantities at a given time instant. It is important to observe that when the number of tokens increases the quantitative behavior obtained applying the stochastic approach tends to that obtained from the ODEs [31]. Hence from the SPN description of the biological phenomenon, the choice of using one of the two approaches (stochastic or deterministic) for studying the behavior of the system is left to the analyst who decides on the basis of the objectives of his/her study. In this paper we use the deterministic approach because it allows a faster and simpler evaluation of the proposed pathway.

### How to use the P-semiflow to reduce the number of ODEs

P-semiflows can be used to reduce the number of ODEs representing the behavior of the system, by identifying those which are redundant. Indeed, as already explained, P-semiflows, can be used to derive the set of places in which the total mass is preserved so that the sum of their corresponding ODEs yields a zero identity. Hence, for each minimal P-semiflow in the model we can select one place belonging to it and re-write its corresponding variable as a linear combination of the other variables in the same P-semiflow. In this way we reduce the number of ODEs of one unit for each minimal P-semiflow present in the system.

## Competing interests

The authors declare that they have no competing interests.

## Authors' contributions

Cordero and Beccuti designed the project and wrote the manuscript. Cordero, Beccuti and Fornari designed models and performed in silico analysis. Lanzardo and Conti generated the biological data. Calogero, Balbo and Cavallo supervised the project and wrote the paper. All authors read and approved the final manuscript.

## Supplementary Material

Additional file 1 **List of reactions**. Click here for file

Additional file 2 **Parameter Values: reaction rates and concentration values**. Click here for file

## Declarations

This article has been published as part of *BMC Bioinformatics *Volume 14 Supplement 6, 2013: Selected articles from the 10th International Conference on Artificial Immune Systems (ICARIS). The full contents of the supplement are available online at http://www.biomedcentral.com/bmcbioinformatics/supplements/14/S6.
