# Supplementary material for: Multi-level model for the investigation of oncoantigen-driven vaccination effect
Source: BMC Bioinformatics. 2013 Apr 17;14(Suppl 6):S11. doi: 10.1186/1471-2105-14-S6-S11 (PMC3633011; doi:10.1186/1471-2105-14-S6-S11)
Supplement: Additional file 1 — List of reactions. [file 1471-2105-14-S6-S11-S1.pdf]

Portion A - ErbB family activation

R1: $$ErbB1 + EGF \underset{k_2}{\overset{k_1}{\rightleftarrows}} ErbB1 : EGF$$

R2: $$ErbB3 + HGF \underset{k_4}{\overset{k_3}{\rightleftarrows}} ErbB3 : HGF$$

R3: $$2 * ErbB2 \underset{k_6}{\overset{k_5}{\rightleftarrows}} ErbB2 : ErbB2$$

R4: $$ErbB1 : EGF + ErbB2 : ErbB2 \underset{k_8}{\overset{k_7}{\rightleftarrows}} ErbB1 : ErbB2$$

R5: $$ErbB3 : HGF + ErbB2 : ErbB2 \underset{k_{10}}{\overset{k_9}{\rightleftarrows}} ErbB3 : ErbB2$$

R6: $$Grb2 + Gab \underset{k_{12}}{\overset{k_{11}}{\rightleftarrows}} Grb2 : Gab$$

R7: $$Grb2 + Sos \underset{k_{14}}{\overset{k_{13}}{\rightleftarrows}} Grb2 : Sos$$

R8: $$Grb2 : Gab + ErbB1 : ErbB2 \underset{k_{16}}{\overset{k_{15}}{\rightleftarrows}} Grb2 : Gab : ErbB1 : ErbB2$$

R9: $$Grb2 : Gab + ErbB3 : ErbB2 \underset{k_{18}}{\overset{k_{17}}{\rightleftarrows}} Grb2 : Gab : ErbB3 : ErbB2$$

R10: $$Grb2 : Gab + ErbB2 : ErbB2 \underset{k_{20}}{\overset{k_{19}}{\rightleftarrows}} Grb2 : Gab : ErbB2 : ErbB2$$

R11: $$Shc + ErbB1 : ErbB2 \underset{k_{22}}{\overset{k_{21}}{\rightleftarrows}} Shc : ErbB1 : ErbB2$$

R12: $$Shc + ErbB3 : ErbB2 \underset{k_{24}}{\overset{k_{23}}{\rightleftarrows}} Shc : ErbB3 : ErbB2$$

R13: $$Shc : ErbB1 : ErbB2 + Grb2 : Sos \underset{k_{26}}{\overset{k_{25}}{\rightleftarrows}} Shc^*$$

R14: $$Shc : ErbB3 : ErbB2 + Grb2 : Sos \underset{k_{28}}{\overset{k_{27}}{\rightleftarrows}} Shc^*$$

R15: $$Shc^* + Ras : GDP \underset{k_{30}}{\overset{k_{29}}{\rightleftarrows}} Shc^* : Ras : GDP$$

R16: $$Shc^* : Ras : GDP \underset{k_{32}}{\overset{k_{31}}{\rightleftarrows}} Shc^* + Ras : GTP$$

R17: $$Shc^* + Ras : GTP \underset{k_{34}}{\overset{k_{33}}{\rightleftarrows}} Shc^* : Ras : GTP$$

R18: $$Shc^* : Ras : GTP \underset{k_{36}}{\overset{k_{35}}{\rightleftarrows}} Shc^* + Ras : GDP$$

Portion B - Activation of Pi3k

*** ErbB2:ErbB2

R:19 $ErbB2 : ErbB2 + Pi3k \overset{k_{37}}{\underset{k_{38}}{\rightleftarrows}} ErbB2 : ErbB2 : Pi3k$

R:20 $ErbB2 : ErbB2 : Pi3k \overset{k_{39}}{\rightarrow} ErbB2 : ErbB2 : Pi3k^*$

R:21 $ErbB2 : ErbB2 : Pi3k^* + Pip2 \overset{k_{40}}{\underset{k_{41}}{\rightleftarrows}} ErbB2 : ErbB2 : Pi3k^* : Pip2$

R:22 $ErbB2 : ErbB2 : Pi3k^* : Pip2 \overset{k_{42}}{\rightarrow} ErbB2 : ErbB2 : Pi3k + Pip3$

*** Grb2:Gab:ErbB2:ErbB2

R23: $Grb2 : Gab : ErbB2 : ErbB2 + Pi3k \overset{k_{43}}{\rightarrow} Grb2 : Gab : ErbB2 : ErbB2 : Pi3k^*$

R24: $Grb2 : Gab : ErbB2 : ErbB2 : Pi3k^* + Pip2 \overset{k_{44}}{\underset{k_{45}}{\rightleftarrows}} Grb2 : Gab : ErbB2 : ErbB2 : Pi3k^* : Pip2$

R25: $Grb2 : Gab : ErbB2 : ErbB2 : Pi3k^* : Pip2 \overset{k_{46}}{\rightarrow} Grb2 : Gab : ErbB2 : ErbB2 + Pi3k + Pip3$

*** Grb2:Gab:ErbB1:ErbB2

R26: $Grb2 : Gab : ErbB1 : ErbB2 + Pi3k \overset{k_{47}}{\rightarrow} Grb2 : Gab : ErbB1 : ErbB2 : Pi3k^*$

R27: $Grb2 : Gab : ErbB1 : ErbB2 : Pi3k^* + Pip2 \overset{k_{48}}{\underset{k_{49}}{\rightleftarrows}} Grb2 : Gab : ErbB1 : ErbB2 : Pi3k^* : Pip2$

R28: $Grb2 : Gab : ErbB1 : ErbB2 : Pi3k^* : Pip2 \overset{k_{50}}{\rightarrow} Grb2 : Gab : ErbB1 : ErbB2 + Pi3k + Pip3$

*** Grb2:Gab:ErbB3:ErbB2

R29: $Grb2 : Gab : ErbB3 : ErbB2 + Pi3k \overset{k_{51}}{\rightarrow} Grb2 : Gab : ErbB3 : ErbB2 : Pi3k^*$

R30: $Grb2 : Gab : ErbB3 : ErbB2 : Pi3k^* + Pip2 \overset{k_{52}}{\underset{k_{53}}{\rightleftarrows}} Grb2 : Gab : ErbB3 : ErbB2 : Pi3k^* : Pip2$

R31: $Grb2 : Gab : ErbB3 : ErbB2 : Pi3k^* : Pip2 \overset{k_{54}}{\rightarrow} Grb2 : Gab : ErbB3 : ErbB2 + Pi3k + Pip3$

*** Tlr*

R32: $Tlr^* + Pi3k \overset{k_{55}}{\rightarrow} Tlr^* : Pi3k^*$

R33: $Tlr^* : Pi3k^* + Pip2 \overset{k_{56}}{\underset{k_{57}}{\rightleftarrows}} Tlr^* : Pi3k^* : Pip2$

R34: $Tlr^* : Pi3k^* : Pip2 \overset{k_{58}}{\rightarrow} Tlr^* + Pi3k + Pip3$

*** Tlr*:Myd88

R35: $Tlr^* : Myd88 + Pi3k \overset{k_{59}}{\rightarrow} Tlr^* : Myd88 : Pi3k^*$

R36: $Tlr^* : Myd88 : Pi3k^* + Pip2 \overset{k_{60}}{\underset{k_{61}}{\rightleftarrows}} Tlr^* : Myd88 : Pi3k^* : Pip2$

R37: $Tlr^* : Myd88 : Pi3k^* : Pip2 \overset{k_{62}}{\rightarrow} Tlr^* : Myd88 + Pi3k + Pip3$

*** RasGTP

R38: $Ras : GTP + Pi3k \overset{k_{63}}{\rightarrow} Ras : GTP : Pi3k^*$

R39: $Ras : GTP : Pi3k^* + Pip2 \overset{k_{64}}{\underset{k_{65}}{\rightleftarrows}} Ras : GTP : Pi3k^* : Pip2$

R40: $Ras : GTP : Pi3k^* : Pip2 \overset{k_{66}}{\rightarrow} Ras : GTP + Pi3k + Pip3$

Pip2 regeneration

R:41 $Pip3 + Pten \overset{k_{67}}{\underset{k_{68}}{\rightleftarrows}} Pip3 : Pten$

R:42 $Pip3 : Pten \overset{k_{69}}{\rightarrow} Pip2 + Pten$

R:43 $Pten + Pip2 \overset{k_{70}}{\underset{k_{71}}{\rightleftarrows}} Pten : Pip2$

R:44 $Pten : Pip2 + Pip3 \overset{k_{72}}{\underset{k_{73}}{\rightleftarrows}} Pten : Pip2 : Pip3$

R:45 $Pten : Pip2 : Pip3 \overset{k_{74}}{\rightarrow} Pten : Pip2 + Pip2$

Portion C - Akt Action

R:46 $$Pip3 + Akt \underset{k_{76}}{\overset{k_{75}}{\rightleftarrows}} Pip3 : Akt$$
R:47 $$Pip3 : Akt + Pdk1 \underset{k_{78}}{\overset{k_{77}}{\rightleftarrows}} Pip3 : AktP : Pdk1$$
R:48 $$Pip3 : AktP : Pdk1 + mTORC2^* \underset{k_{80}}{\overset{k_{79}}{\rightleftarrows}} Pip3 : AktP : Pdk1 : mTORC2^*$$
R:49 $$Pip3 : AktP : Pdk1 : mTORC2^* \overset{k_{81}}{\rightarrow} Pip3 : Akt^* : Pdk1 + mTORC2^*$$

NF-kb

R:50 $$Pip3 : Akt^* : Pdk1 + IKK\alpha - \beta \overset{k_{82}}{\rightarrow} Pip3 : Akt^* : Pdk1 + IKK\alpha - \beta P$$
R:51 $$NFkB + IKB \overset{k_{83}}{\rightarrow} NFkB : IKB$$
R:52 $$IKK\alpha\beta P + NFkB : IKB \overset{k_{84}}{\rightarrow} NFkB + IKBP + IKK\alpha - \beta$$
R:53 $$IKBP \overset{k_{85}}{\rightarrow} IKB$$
R:54 $$TLR^* : MyD88 : IRAK : TRAF6 : TAKI + IKK\alpha - \beta \overset{k_{91}}{\rightarrow} IKK\alpha - \beta P$$

Cyclin D1

R:55 $$Pip3 : Akt^* : Pdk1 + PKC \overset{k_{86}}{\rightarrow} Pip3 : Akt^* : Pdk1 + PKC^*$$
R:56 $$PKC^* + Gsk3 \overset{k_{87}}{\rightarrow} Gsk3P + PKC$$
R:57 $$Pip3 : Akt^* : Pdk1 + Gsk3 \overset{k_{88}}{\rightarrow} Pip3 : Akt^* : Pdk1 + Gsk3P$$
R:58 $$Gsk3P + CyclinD1P \overset{k_{89}}{\rightarrow} CyclinD1$$
R:59 $$CyclinD1 \overset{k_{90}}{\rightarrow} CyclinD1P$$

BAD

R:60 $$Pip3 : Akt^* : Pdk1 + Bad \overset{k_{92}}{\rightarrow} Pip3 : Akt^* : Pdk1 + BadP$$
R:61 $$BadP \overset{k_{93}}{\rightarrow} Bad$$

Portion D - Akt and TCS

R:62 $$Pip3 : Akt^* : Pdk1 + Tsc1 - Tsc2 \overset{k_{94}}{\rightarrow} Pip3 : Akt^* : Pdk1 + Tsc1P - Tsc2P$$
R:63 $$Tsc1P - Tsc2P + P14 - 3 - 3 \overset{k_{95}}{\rightarrow} Tsc1P - Tsc2P + Tsc1P - Tsc2P : 14 - 3 - 3$$
R:64 $$Tsc1 - Tsc2 + RhebGTP \overset{k_{96}}{\rightarrow} Tsc1 - Tsc2^* + RhebGDP$$
R:65 $$Tsc1 - Tsc2^* + mTORC2 \overset{k_{97}}{\rightarrow} mTORC2^* + Tsc1 - Tsc2$$
R:66 $$RhebGTP + mTORC1 \overset{k_{98}}{\rightarrow} mTORC1^*$$
R:67 $$mTORC1^* + P14 - 3 - 3 \overset{k_{99}}{\rightarrow} mTORC1^* + Prot14$$
R:68 $$mTORC1^* + S6K \overset{k_{100}}{\rightarrow} mTORC1^* + S6KP$$
R:69 $$S6RP + S6KP \overset{k_{101}}{\rightarrow} S6RPP + S6KP$$
R:70 $$eIF4G + S6KP \overset{k_{102}}{\rightarrow} eIF4GP + S6KP$$
R:71 $$eIF4B + S6KP \overset{k_{103}}{\rightarrow} eIF4BP + S6KP$$
R:72 $$RhebGDP \overset{k_{104}}{\rightarrow} RhebGTP$$
R:73 $$PP2A + Gsk3P \overset{k_{105}}{\rightarrow} PP2A + Gsk3$$
R:74 $$PP2A + S6KP \overset{k_{106}}{\rightarrow} PP2A + S6K$$
R:75 $$Pip3 : Akt^* : Pdk1 + PP2A \underset{k_{108}}{\overset{k_{107}}{\rightleftarrows}} Pip3 : Akt^* : Pdk1 : PP2A$$
R:76 $$Pip3 : Akt^* : Pdk1 : PP2A \overset{k_{109}}{\rightarrow} Pip3 : Akt : Pdk1 + PP2A$$
R:77 $$PP2A + mTORC1^* \underset{k_{125}}{\overset{k_{111}}{\rightleftarrows}} PP2ADegradation + mTORC1^*$$

Portion E - Tlr receptor action

**R:78**	$Tlr^* + Myd88$	$\underset{k_{113}}{\overset{k_{112}}{\rightleftarrows}}$	$Tlr^* : Myd88$
**R:79**	$Tlr^* : Myd88 + IRAK$	$\underset{k_{115}}{\overset{k_{114}}{\rightleftarrows}}$	$Tlr^* : Myd88 : IRAK$
**R:80**	$Tlr^* : Myd88 : IRAK + Traf6$	$\underset{k_{117}}{\overset{k_{116}}{\rightleftarrows}}$	$Tlr^* : Myd88 : IRAK : Traf6$
**R:81**	$Tlr^* : Myd88 : IRAK : Traf6 + Ask1$	$\overset{k_{118}}{\rightarrow}$	$Tlr^* : Myd88 : IRAK : Traf6 : Ask1$
**R:82**	$Tlr^* : Myd88 : IRAK : Traf6 : Ask1 + Jnk$	$\overset{k_{119}}{\rightarrow}$	$Tlr^* : Myd88 : IRAK : Traf6 : Ask1 + JnkP$
**R:83**	$Pip3 : Akt^* : Pdk1 + Ask1$	$\overset{k_{120}}{\rightarrow}$	$Ask1P + Pip3 : Akt^* : Pdk1$
**R:84**	$Tlr^* : Myd88 : IRAK : Traf6 + Tp12$	$\overset{k_{121}}{\rightarrow}$	$Tlr^* : Myd88 : IRAK : Traf6 : Tp12$
**R:85**	$Tlr^* : Myd88 : IRAK : Traf6 + Tak1$	$\overset{k_{122}}{\rightarrow}$	$Tlr^* : Myd88 : IRAK : Traf6 : Tak1$
**R:86**	$GF2 + Tlr$	$\underset{k_{124}}{\overset{k_{123}}{\rightleftarrows}}$	Tlr^*
