# Supplementary material for: Multi-level model for the investigation of oncoantigen-driven vaccination effect
Source: BMC Bioinformatics. 2013 Apr 17;14(Suppl 6):S11. doi: 10.1186/1471-2105-14-S6-S11 (PMC3633011; doi:10.1186/1471-2105-14-S6-S11)
Supplement: Additional file 2 — Parameter Values: reaction rates and concentration values. [file 1471-2105-14-S6-S11-S2.pdf]

Concentrations

Substrate	Concentration
Akt	4440
ASK1	20
ASK1P	0
B1B2GaGrPi3k	0
B1B2GaGrPiP2	0
B3B2GrGaPi3k	0
B3B2GrGaPiP2	0
Bad	50
BadP	0
BadP:Pr14	0
CycD1P	600
CycD1	0
eIF4B	50
eIF4BP	0
eIF4G	50
eIF4GP	0
ERBB1	524
ERBB1:EGF	0
ERBB1:ERBB2	0
ERBB1ERBB2GaGr	0
ERBB2	1580
ERBB3	684
ERBB3:ERBB2	0
ERBB3:HG	0
ERBB3ERBB2GaGr	0
Gab	43
GabGrb2	0
Grb2	82
GrbSos	0
Gsk3	50
Gsk3P	0
E2*:Gr:Gab:Pik*	0
E2*:Gr:Gab:Pk*:P2	0
ERBB2*:Gr:Gab	0
ERBB2*:Gr:Gab:So	0
Shc:RasGT	0
Shc:RasGD	0
ERBB2*:Pi3k	0
ERBB2*:Pi3k*	0
ERBB2*:Pi3k*:P2	0
IKB	50
IKBP	0
IKKab	5000
IKKaPb	0
IRAK	80
JNK	50
JNKP	0
MyD88	80
NFkB	0
NFkB:IKB	50
P2	1960
P2:Pten	0
P3	0
P3:Akt	0
P3:Akt*:Pdk1	0

P3:Akt*:Pdk1:PP	0
P3:Akt:Pdk1	0
P3:AktP:Pdk1	0
P3:AktP:Pdk1:TO2*	0
P3:Pten	0
Pdk1	4440
Pi3k	46
PKC	50
PKC*	0
PP	5000
PPDegr	0
Pr14	50
Pr14P	0
Pten	46
Pten:P2:P3	0
Ras:GD	95
Ras:GT	0
Ras:GT:Pi3k*	0
Ras:GT:Pi3k*:P2	0
RhebGD	50
RhebGT	0
S6K	50
S6KP	0
Shc	11
Sos	82
TAK1	90
TLR	see paper
TLR*	0
TLR:MyD88	0
TLR:MyD88:IRAK	0
TLR:MyD88:IRAK:T	0
TLR:MyD88:IRAK:T	0
TLR:MyD88:Pi3k*	0
TLR:MyD88:Pi3k*:P	0
TLR:MyDD88:IRAK:	0
TLR:MyDD88:IRAK:	0
TLR:Pi3k*	0
TLR:Pi3k*:P2	0
TO1	50
TO1*	0
TO2	50
TO2*	0
Tpl2	20
TRAF6	80
Tsc1PTsc2P	0
Tsc1PTsc2P:Pr14	0
Tsc1Tsc2	0
Tsc1Tsc2*	50

Transition	Value
k1	1
k2	0.0056
k3	0.006
k4	0.0056
k5	0.172
k6	0.501
k7	0.228
k8	2.662
k9	0.228
k10	2.662
k11	0.005
k12	7.049
k13	0.019
k14	3.105
k15	0.09
k16	2.336
k17	1
k18	3.696
k19	0.001
k20	3.696
k21	1
k22	1
k23	1
k24	1
k25	0.048
k26	0.676
k27	0.048
k28	15
k29	22.175;
k30	276
k31	12
k32	276
k33	276
k34	22.175
k35	276
k36	22.175
k37	0.015
k38	4.423
k39	0.0005
k40	0.005
k41	0.01
k42	0.008
k43	0.0005
k44	0.005
K45	0.01
k46	0.1
k47	0.0005
k48	0.005
k49	0.01
k50	0.008
k51	0.0005
k52	0.005
k53	0.01
k54	0.1
k55	0.0005
k56	0.005

k57	0.01
k58	0.008
k59	0.0005
k60	0.005
k61	0.01
k62	0.008
k63	0.0005
k64	0.005
k65	0.01
k66	0.008
k67	3
k68	3
k69	16.833
k70	0.01
k71	1
k72	3
k73	1
k74	16.833
k75	24.605
k76	147.62
k77	24.605
k78	147.62
k79	24.605
k80	147.62
k81	24.605
k82	1
k83	1
k84	1
k85	1
k86	1
k87	1
k88	1
k89	1
k90	1
k91	0.0002
k92	1
k93	1
k94	1
k95	1
k96	1
k97	1
k98	1
k99	1
k100	1
k101	1
k102	1
k103	1
k104	1
k105	1
k106	1
k107	1
k108	0.0003
k109	1
k111	0.0001
k125	0.03
k112	1
k113	4

k114	1
k115	4
k116	1
k117	4
k118	1
k119	1
k120	1
k121	1
k122	1
k123	1
k124	0.0056
